# Supplementary material for: Structure of a cyanobacterial photosystem I surrounded by octadecameric IsiA antenna proteins
Source: Commun Biol. 2020 May 11;3:232. doi: 10.1038/s42003-020-0949-6 (PMC7214436; doi:10.1038/s42003-020-0949-6)
Supplement: Supplementary file 1 — Supplementary Information [file 42003_2020_949_MOESM1_ESM.pdf]

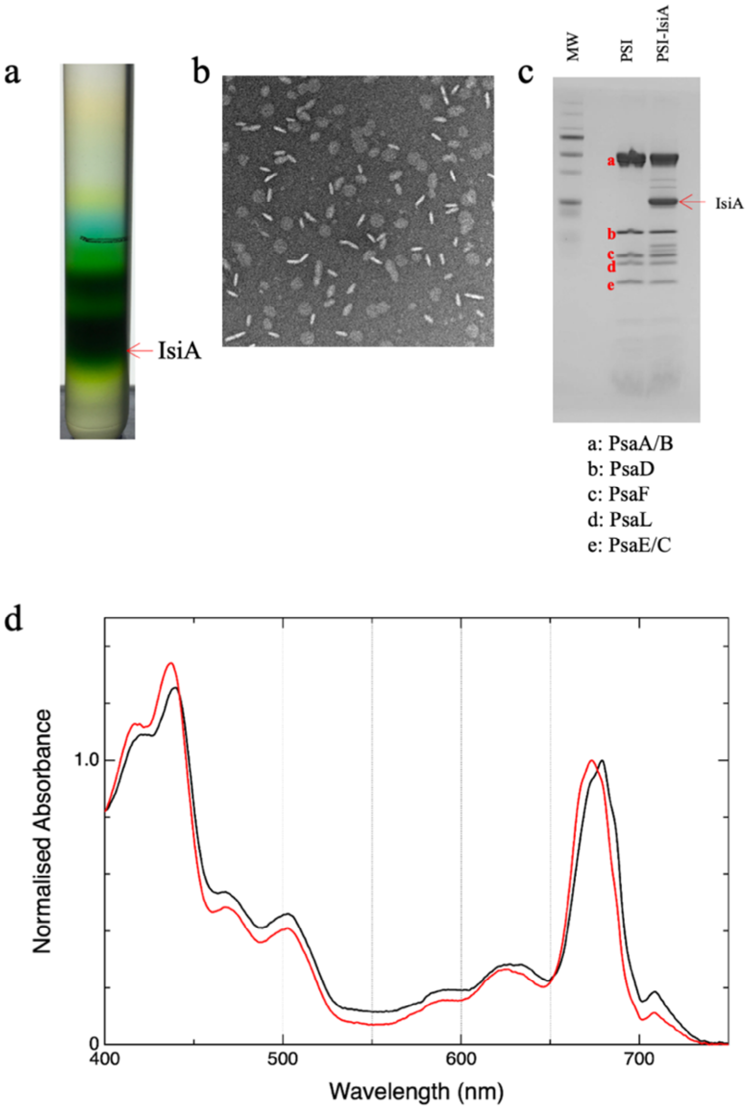

Supplementary Fig. 1. Preparation of PSI-IsiA. a, Trehalose density gradient centrifugation of solubilized thylakoid membranes. The PSI-IsiA containing faction was indicated by a red arrow. **b,** An electron micrograph of negatively stained PSI-IsiA. **c,** SDS-PAGE analysis of the purified PSI-IsiA and PSI core obtained from the density gradient centrifugation. The IsiA band was indicated by a red arrow. **d,** Absorption spectra of PSI-IsiA (red line) and PSI trimer (black line) measured at 77 K.

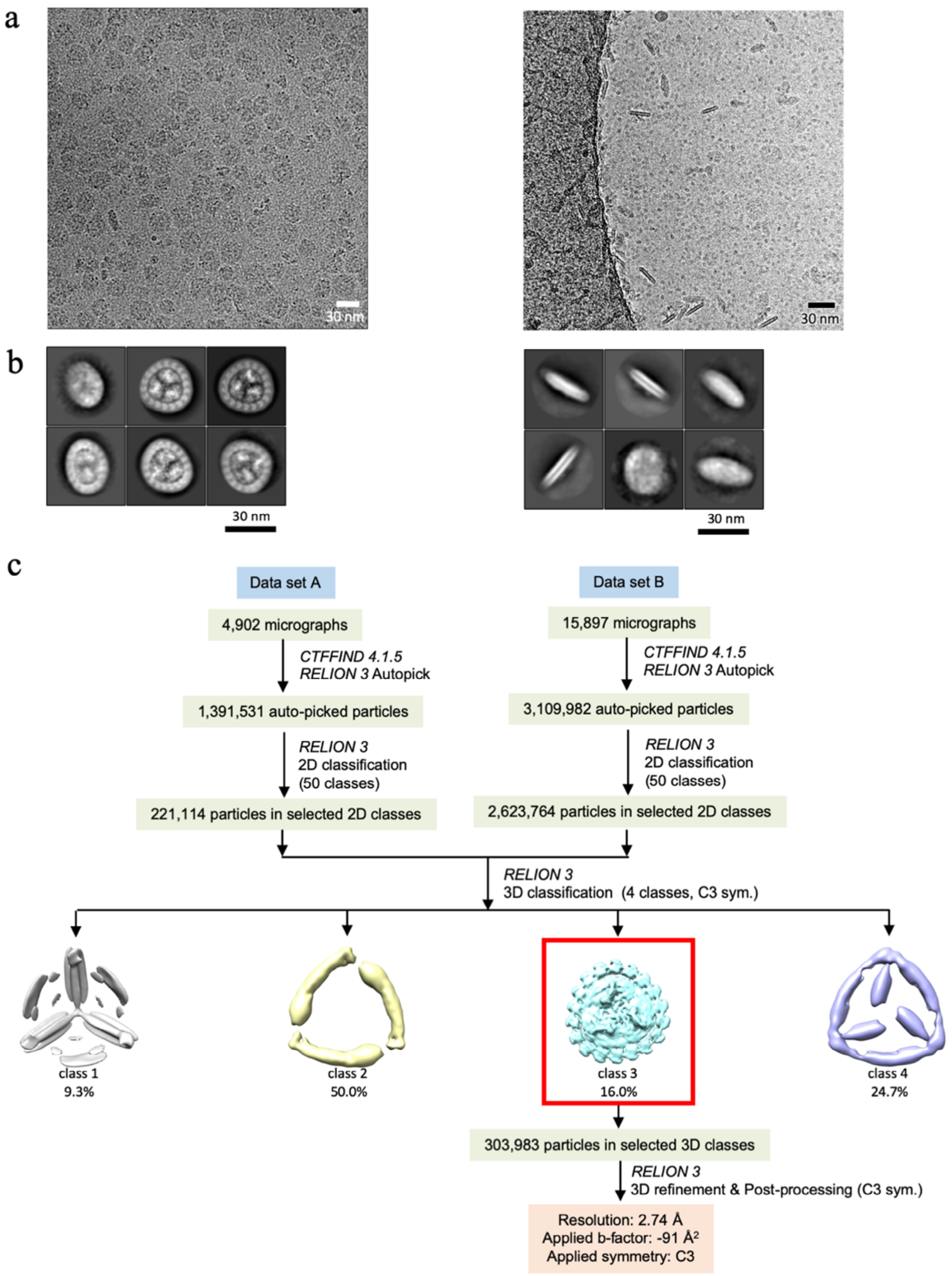

Supplementary Fig. 2. Single particle cryo-EM analysis of PSI-IsiA. a, A representative cryo-EM micrograph of purified PSI-IsiA. **b,** Representative reference-free 2D-classes. **c,** Processing procedure for the cryo-EM data of PSI-IsiA.

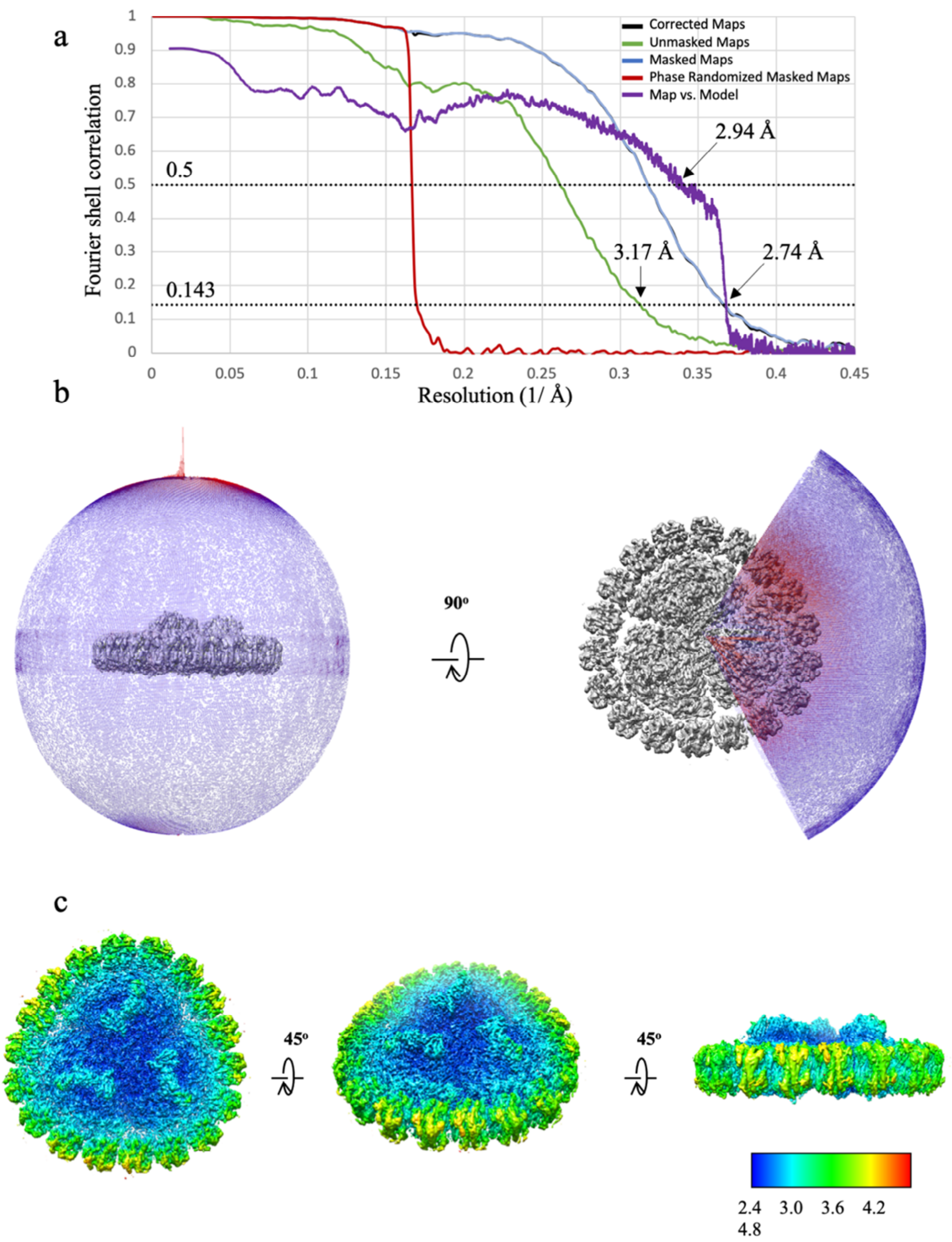

Supplementary Fig. 3. Evaluation of the resolution of the cryo-EM map of PSI-IsiA. **a,** The FSC curve calculated between independently refined half maps of the reconstruction. **b,** The Eular angle distributions. **c,** Local resolution maps of the PSI-IsiA supercomplex.

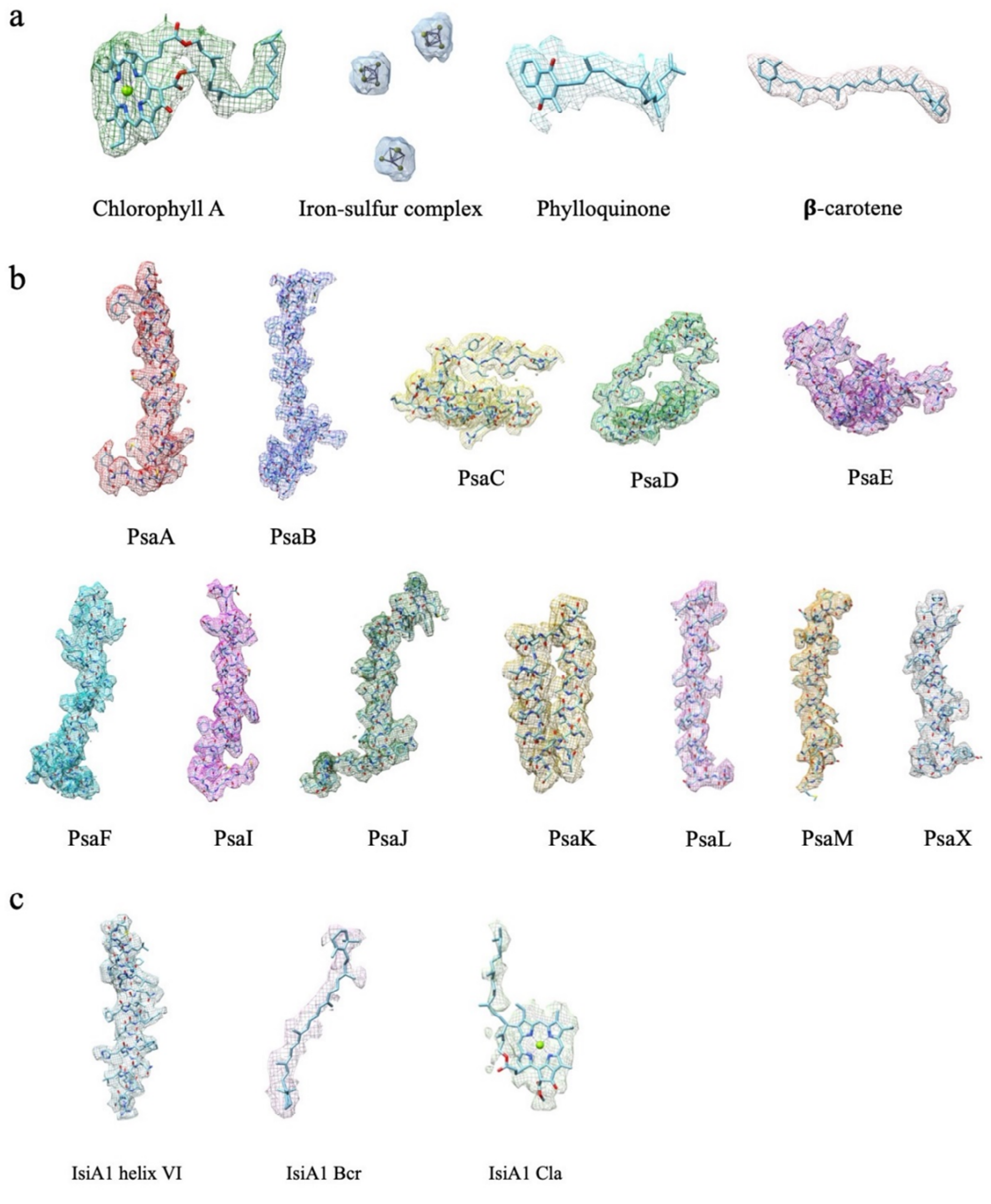

Supplementary Fig. 4. Cryo-EM maps and molecular models for representative cofactors and each subunit of the PSI core and IsiA. a, Cryo-EM maps for the representative cofactors for PSI. **b,** Cryo-EM maps for each subunit of the PSI core. **c,** Cryo-EM map for IsiA.

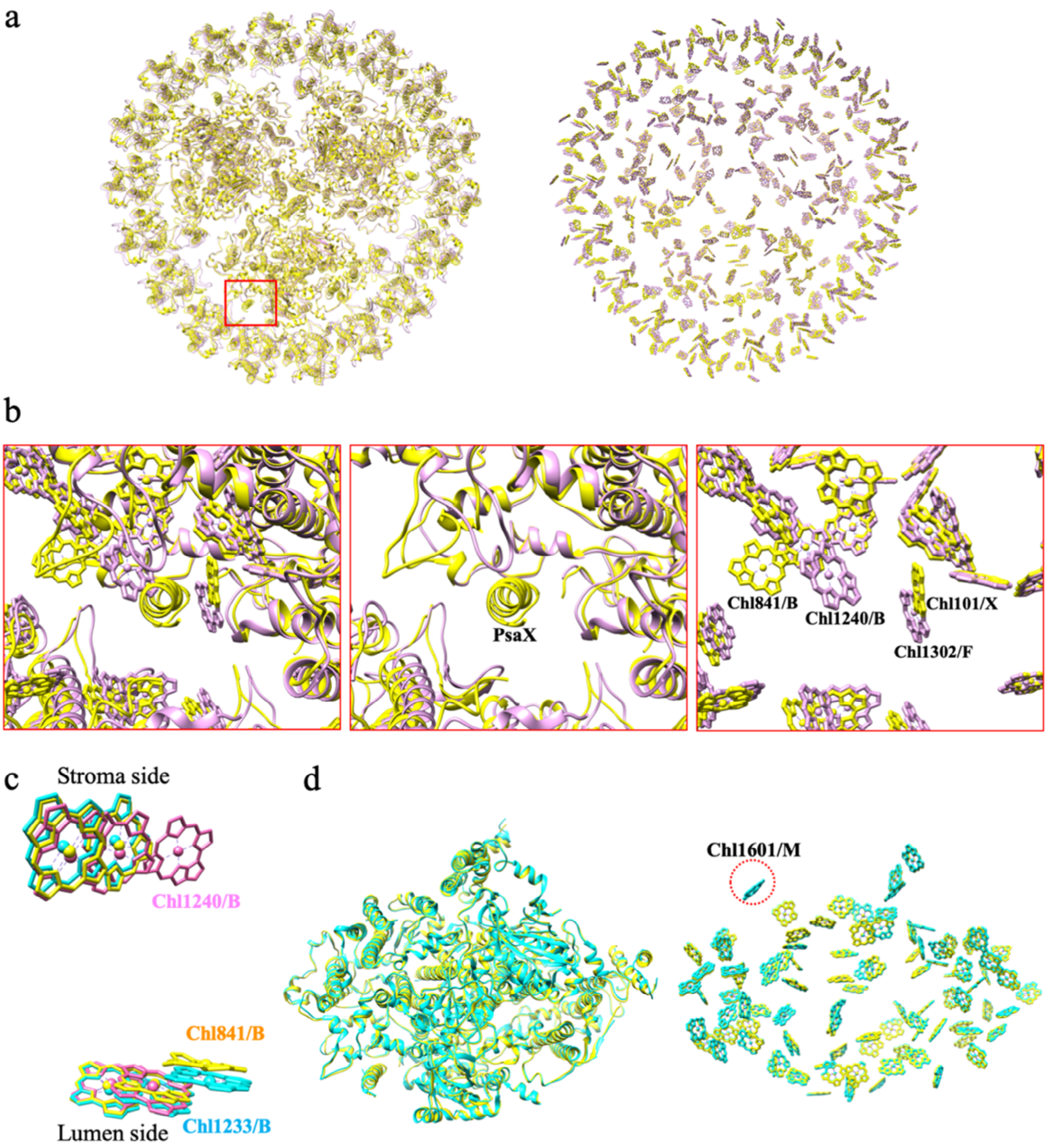

Supplementary Fig. 5. Comparison between the structures of T_PSI-IsiA and S_PSI-IsiA. a, Superposition of T_PSI-IsiA (yellow) and S_PSI-IsiA (pink). Left panel: Superposition of the protein structures. Right panel: Superposition of Chls. A red square indicates the region enlarged in panel b. **b,** The differences between T_PSI-IsiA and S_PSI-IsiA around the PsaX region of. **c**, Superimposition of tripled Chls of *T. vulucanus* (yellow), *T. elongatus* (1JB0) (cyan) and *S.sp*. PCC 6803 (6NWA) (pink). **d**, Superimposition of PSI core of *T. vulcanus* (yellow) and *T. elongatus* (1JB0) (cyan). Left panel: Superposition of the protein structures. Right panel: Superposition of Chls. A red circle indicates the difference between the two PSIs.

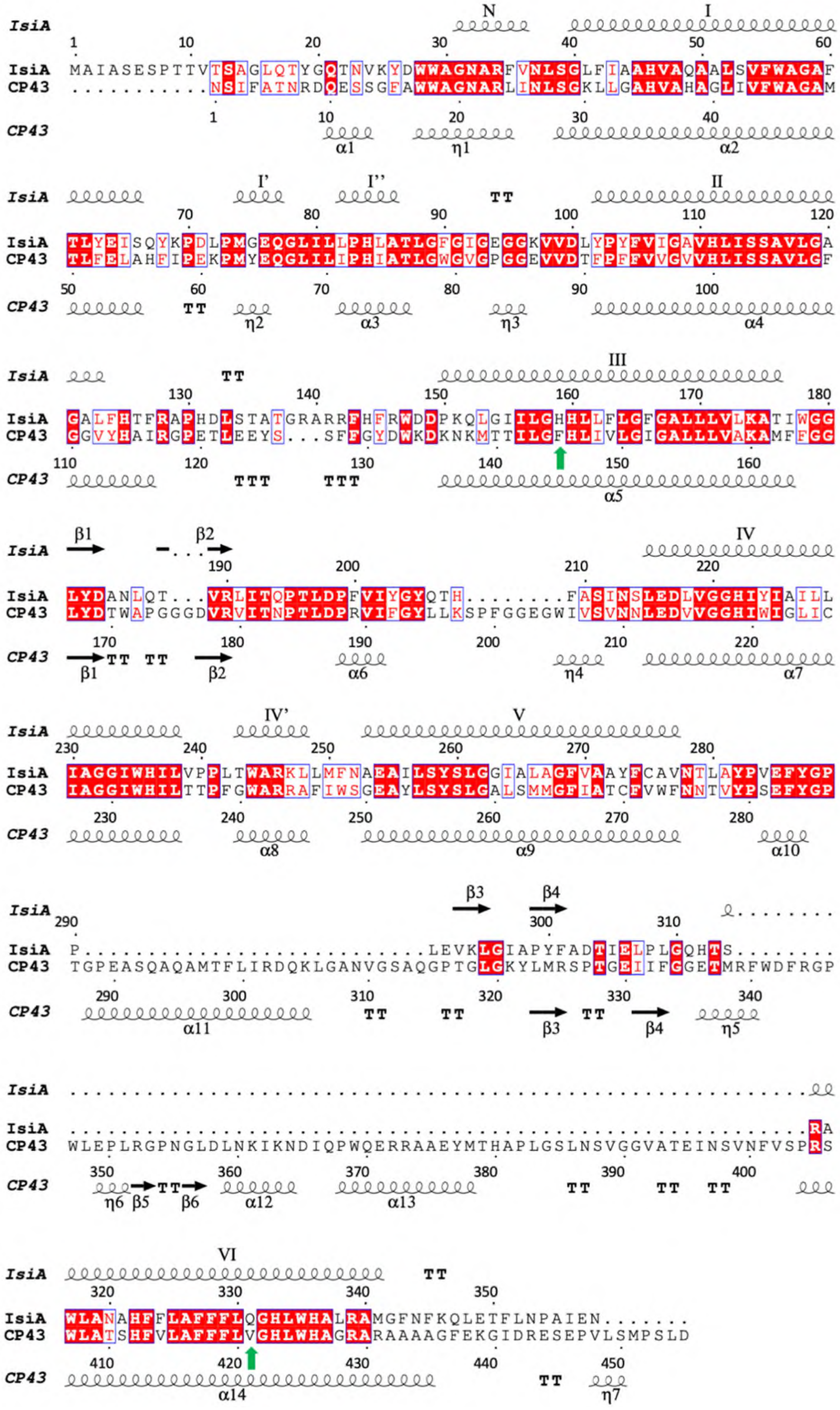
IsiA MAIASESPTTVTSAGLQTYGQTNVKYDWWAGNARFVNLSGLFIAAHVAQAALSVFWAGAF
CP43 ...........NSIFATNRDQESSGFAWWAGNARLINLSGKLLGAHVAHAGLIVFWAGAM
IsiA TLYEISQYKPDLPMGEQGLILLPHLATLGFGIGEGGKVVDLYPYFVIGAVHLISSAVLGA
CP43 TLFELAHFIPEKPMYEQGLILIPHIATLGWGVGPGGEVVDTFPFFVVGVVHLISSAVLGF
IsiA GALFHTFRAPHDLSTATGRARRFHFRWDDPKQLGIILGHLLFLGFGALLLVLKATIWGG
CP43 GGVYHAIRGPETLEEYS...SFFGYDWKDKNKMTTILGFHLIVLGIGALLLVAKAMFFGG
IsiA LYDANLQT...VRLITQPTLDPFVIYGYQTH........FASINSLEDLVGGHIYIAILL
CP43 LYDTWAPGGGDVRVITNPTLDPRVIFGYLLKSPFGGEGWIVSVNNLEDVVGGHIWIGLIC
IsiA IAGGIWHILVPPLTWARKLLMFNAEAILSYSLGGIALAGFVAAYFCAVNTLAYPVEFYGP
CP43 IAGGIWHILTTPFGWARRAFIWSGEAYLSYSLGALSMMGFIATCFVWFNNTVYPSEFYGP
IsiA P........................LEVKLGIAPYFADTIELPLGQHTS.........
CP43 TGPEASQAQAMTFLIRDQKLGANVGSAQGPTGLGKYLMRSPTGEIIFGGETMRFWDFRGP
IsiA ....RA
CP43 WLEPLRGPNGLDLNKIKNDIQPWQERRAAEYMTHAPLGSLNSVGGVATEINSVNFVSPRS
IsiA WLANAHFFLAFFFLQGHLWHALRAMGFNFKQLETFLNPAIEN.......
CP43 WLATSHFVLAFFFLVGHLWHAGRARAAAAGFEKGIDRESEPVLSMPSLD

Supplementary Fig. 6. Alignment of amino acid sequences between IsiA and the CP43 of PSII. The sequence of CP43 is taken from *T. vulucanus* (PDB ID: 3WU2) and aligned by Clustal W[1], and the figure was drawn by ESPript[2]. The secondary-structural elements are indicated as spirals (helix), arrows (sheet) and the letter T (turn). White letters on a red background indicate identical amino acids, whereas red letters in blue boxes indicate similar residues. Gaps are shown as dotted lines, and green arrows indicate the amino acid ligands for Chls present in IsiA.

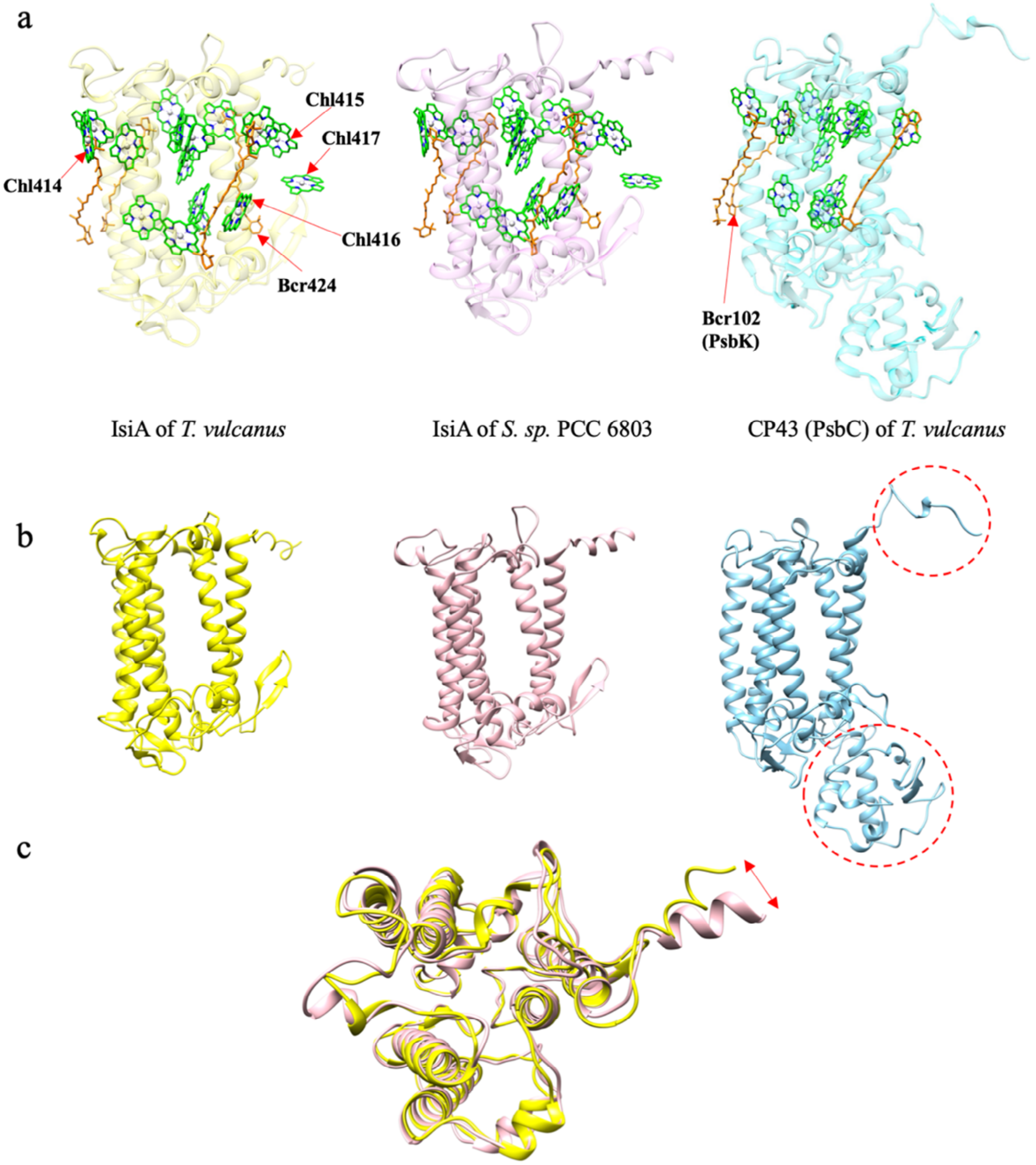

Supplementary Fig. 7. Comparison between the structures of IsiAs and CP43. a, Distribution of pigments in IsiA of *T. vulcanus* (yellow), IsiA of *S.sp*. PCC 6803 (Pink) and CP43 (blue) of PSII (PDB ID: 3WU2). Bcr102 in CP43 has been assigned to PsbK. The red arrows indicate pigments that are characteristic each of the subunits. **b,** Protein structures of the two subunits depicted in ribbon diagrams. Red circles indicate the apparent different regions between IsiA and CP43. c, Superimposition of IsiA4 of *T. vulcanus* and IsiAY of *S.sp*. PCC 6803.

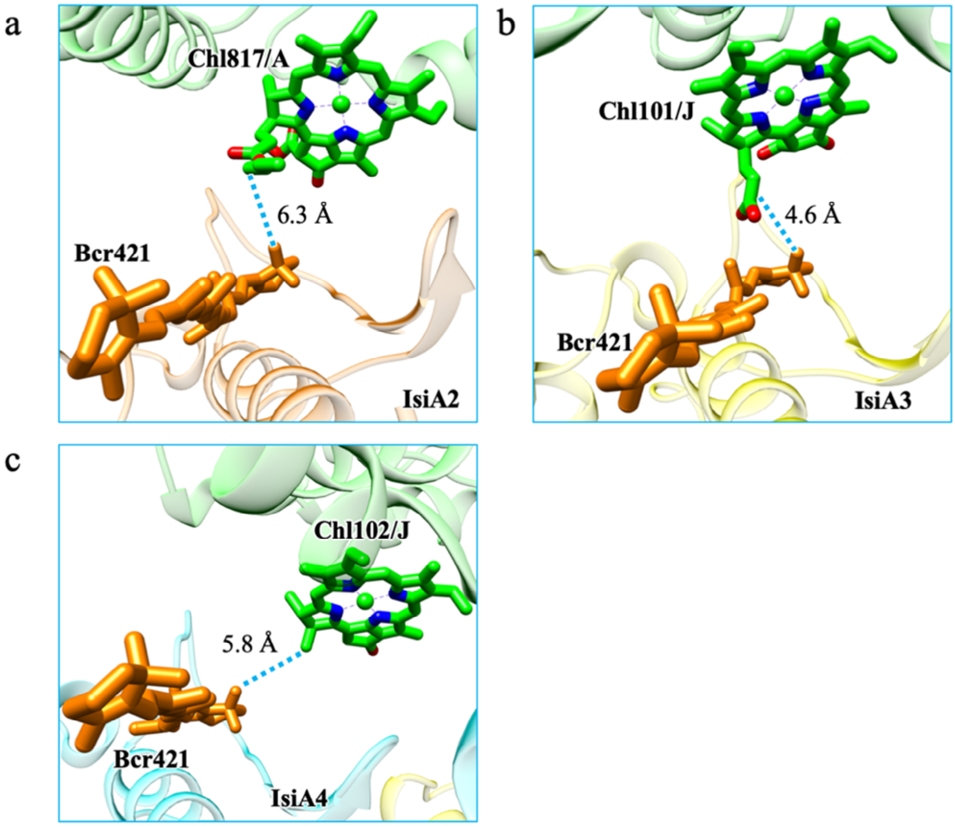

Supplementary Fig. 8. Bcr-Chl interactions between PSI and IsiA. a, Bcr-Chl interactions between PSI and IsiA2. **b,** Bcr-Chl interactions between PSI and IsiA3. **c,** Bcr-Chl interactions between PSI and IsiA4. Interactions were indicated by dashed lines.

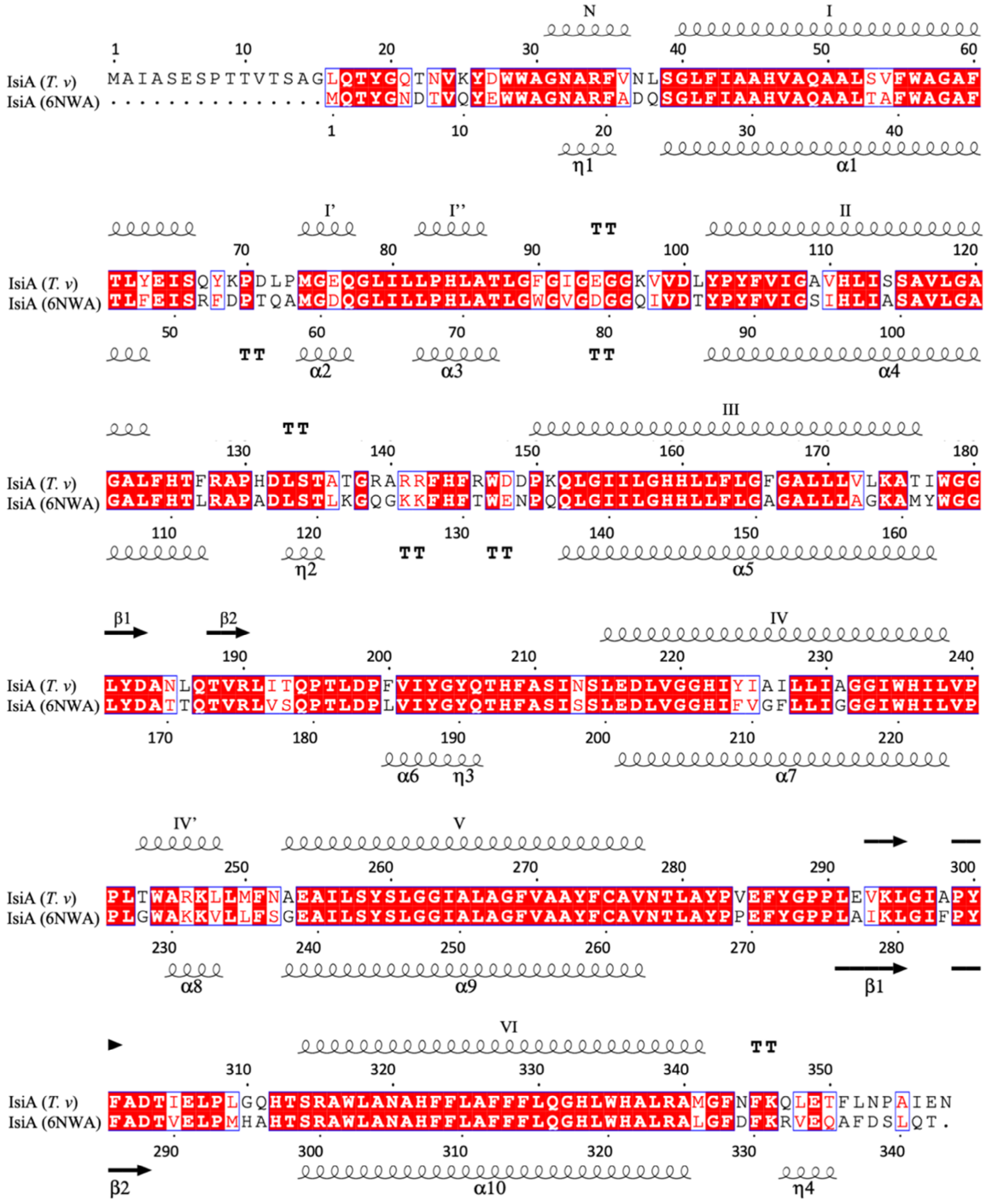

Supplementary Fig. 9. Alignment of amino acid sequences between IsiA from *T. vulucanus* and IsiA from *S.sp.* PCC 6803. The sequences were aligned by Clustal W and the figure was drawn by ESPript[1,2]. The secondary-structural elements are indicated as a spirals (helix), arrows (sheet) and T (turn). White letters on a red background indicate identical amino acids, whereas red letters in blue boxes indicate similar residues. Gaps are shown as dotted lines.

Supplementary Table 1. Cryo-EM data collection, refinement and validation statistics

	PSI isiA<br>(EMDB-9908)<br>(PDB 6K33)
**Data collection and processing**	
Magnification	59,000
Voltage (kV)	300
Electron exposure (e-/Å^2)	50
Defocus range (μm)	-1.5 to -3.5
Pixel size (Å)	1.113
Symmetry imposed	C3
Initial particle images (no.)	4,501,513
Final particle images (no.)	303,983
Map resolution (Å)	2.74
FSC threshold	0.143
Map resolution range (Å)	2.4 to 4.8
**Refinement**	
Initial model used (PDB code)	1JB0
Model resolution (Å)	2.94
FSC threshold	0.5
Map sharpening *B* factor (Å^2)	-91.8
Model composition	
Non-hydrogen atoms	137,634
Protein residues	12,681
Ligands	38,664
B factors (Å^2)	
Protein	55.9
Ligand	60.1
R.m.s. deviations	
Bond lengths (Å)	0.011
Bond angles (°)	1.317
Validation	
MolProbity score	2.64
Clashscore	12.16

Poor rotamers (%)	6.41
Ramachandran plot	
Favored (%)	93.46
Allowed (%)	6.08
Disallowed (%)	0.45

Supplementary Table 2. Cofactors found in each monomer unit of the PSI-IsiA supercomplex.

Proteins	Chls	Bcrs	Quinones	Lipids	Others
**PsaA**	45 Chl *a*<br>1 Chl *a´*	6 Bcr	1 naphthoquinone	2 LHG	1 Fe-S cluster
**PsaB**	39 Chl *a*	7 Bcr	1 naphthoquinone	1 LHG<br>1LMG	
**PsaC**					2 Fe-S cluster
**PsaD**					
**PsaE**					
**PsaF**	1 Chl *a*	1 Bcr			
**PsaI**		2 Bcr			
**PsaJ**	2 Chl *a*	3 Bcr			
**PsaK**	1 Chl *a*				
**PsaL**	3 Chl *a*	2 Bcr			1 Ca
**PsaM**		1 Bcr			
**PsaX**	1 Chl *a*				
**PSI monomer**	93	22	2	4	4
**PSI trimer**	279	66	6	12	12
**isiA**	17 Chl *a*	4 Bcr			
**isiA total**	306	72			
**Total**	585	138	6	12	12

Bcr, *β*-carotene; LMG, distearoylmonogalactosyl diglyceride; LHG, dipalmitoylphosphatidylglycerol.

Supplementary Table 3. Pigment binding sites and their ligands in IsiA monomers from *T. vulcanus* and *Synechocystis* sp. PCC 6803 and CP43.

	**IsiA1-6**		**IsiA (6NWA)**		**CP43**
**Chls**		No.		No.	
**401**	Chl *a* (H222)	505	Chl *a* (H207)	501	Chl *a* (H227)
**402**	Chl *a* (H322)	506	Chl *a* (H307)	502	Chl *a* (H430)
**403**	Chl *a* (H111)	504	Chl *a* (H96)	503	Chl *a* (H118)
**404**	Chl *a* (-)	514	Chl *a* (H_2O)	504	Chl *a* (-)
**405**	Chl *a* (H333)	515	Chl *a* (H318)	505	Chl *a* (H441)
**406**	Chl *a* (H236)	516	Chl *a* (H221)	506	Chl *a* (H251)
**407**	Chl *a* (-)	510	Chl *a* (H_2O)	507	Chl *a* (-)
**408**	Chl *a* (H336)	511	Chl *a* (H321)	508	Chl *a* (H444)
**409**	Chl *a* (H46)	509	Chl *a* (H31)	509	Chl *a* (H53)
**410**	Chl *a* (Q49)	507	Chl *a* (Q34)	510	Chl *a* (H56)
**411**	Chl *a* (N32)	512	Chl *a* (N17)	511	Chl *a* (N39)
**412**	Chl *a* (H160)	502	Chl *a* (H145)	512	Chl *a* (H164)
**413**	Chl *a* (H125)	503	Chl *a* (H110)	513	Chl *a* (H132)
**414**	Chl *a* (H159)	501	Chl *a* (H144)		
**415**	Chl *a* (Q331)	508	Chl *a* (N316)		
**416**	Chl *a* (Q206)	513	Chl *a* (Q191)		
**417**	Chl *a* (I297) *	517	Chl *a* (I282) *		
**Bcrs**		No.		No.	
**421**	Bcr	602	Bcr	514	Bcr
**422**	Bcr	603	Bcr	515	Bcr
**423**	Bcr	601	Bcr	102 (PsbK)	Bcr
**424**	Bcr	604	Bcr		

*Coordinated by backbone carbonyls.

Supplementary Table 4. Buried surface areas, Mg-Mg distances (D), dipole orientation factors (κ^2), Förster energy transfer rate (K_{DA}), lifetime (τ) and Half-life ($t_{1/2}$) between Chls from adjacent IsiA subunits.

IsiAs	Buried surface area ($Å^2$)		Mg to Mg distances between IsiAs: Chlorophylls		D (Å)	κ^2	K_{DA} (ps^{-1})	τ (ps)*	$t_{1/2}$ (ps)*
**IsiA1**	IsiA2	1,395	404 (1)	415 (2)	23.67	1.49	0.05	21.14	14.65
			411 (1)	405 (2)	20.99	1.51	0.10	10.09	6.99
			412 (1)	405 (2)	20.30	0.76	0.06	16.41	11.37
			413 (1)	405 (2)	13.01	0.49	0.57	1.76	1.22
			410 (1)	417 (2)	17.87	0.36	0.06	16.06	11.13
**IsiA2**	IsiA3	1,421	404 (2)	415 (3)	23.97	1.50	0.04	22.66	15.70
			411 (2)	405 (3)	20.79	1.42	0.10	10.14	7.03
			412 (2)	405 (3)	20.66	0.74	0.05	18.82	13.04
			413 (2)	405 (3)	13.12	0.42	0.46	2.18	1.51
			410 (2)	417 (3)	16.87	0.35	0.09	11.82	8.19
**IsiA3**	IsiA4	1,353	404 (3)	415 (4)	22.92	1.61	0.06	16.09	11.15
			404 (3)	417 (4)	14.27	0.33	0.22	4.64	3.22
			411 (3)	405 (4)	21.13	1.47	0.09	10.86	7.53
			412 (3)	405 (4)	21.63	0.76	0.04	24.30	16.84
			413 (3)	405 (4)	14.15	0.32	0.22	4.49	3.11
			410 (3)	417 (4)	17.12	0.52	0.11	8.74	6.06
**IsiA4**	IsiA5	1,412	404 (4)	415 (5)	22.84	1.61	0.06	15.83	10.97
			404 (4)	417 (5)	14.15	0.28	0.19	5.20	3.61
			411 (4)	405 (5)	20.51	1.41	0.11	9.43	6.54
			412 (4)	405 (5)	21.32	0.81	0.05	20.85	14.45
			413 (4)	405 (5)	13.85	0.43	0.34	2.92	2.02
			410 (4)	417 (5)	16.98	0.43	0.10	10.00	6.93
**IsiA5**	IsiA6	1,327	404 (5)	415 (6)	24.04	1.49	0.04	23.18	16.06
			411 (5)	405 (6)	20.53	1.38	0.10	9.70	6.72
			412 (5)	405 (6)	20.39	0.83	0.06	15.57	10.79
			413 (5)	405 (6)	13.00	0.60	0.70	1.44	0.10
			410 (5)	417 (6)	18.52	0.25	0.04	28.41	19.69
**IsiA6**	IsiA1	1,152	411 (6)	405 (1)	20.63	1.38	0.10	9.99	6.92
			412 (6)	405 (1)	19.96	0.84	0.07	13.48	9.34
			413 (6)	405 (1)	12.28	0.58	0.94	1.07	0.74

*The lifetime (τ) and Half-life ($t_{1/2}$) were defined as $\tau = 1/K_{DA}$ and $t_{1/2} = \ln2/K_{DA}$, respectively.

Supplementary Table 5. Buried surface areas, Mg-Mg distances (D), dipole orientation factors (κ^2), Förster energy transfer rate (K_{DA}), lifetime (τ) and half-life ($t_{1/2}$) between Chls in PSI and IsiAs.

IsiAs	Buried surface areas between PSI-IsiAs ($Å^2$)	Interactions between PSI and IsiAs: Residues PSI	IsiA	D (Å)	Mg to Mg distances between PSI and IsiAs: Chlorophylls PSI	IsiA	D (Å)	κ^2	K_{DA} (ps^{-1})	τ (ps)*	$t_{1/2}$ (ps)*
**IsiA1**	267.4	L43(K)	D27	4.0	845(A)	417	13.71	0.43	0.36	2.75	1.91
		L43(K)	W29	-	845(A)	415	15.48	0.21	0.09	11.66	8.08
		D257(A)	G310	4.3	845(A)	408	20.94	1.33	0.09	11.36	7.87
**IsiA2**	103.8	W177(A)	W29	3.8	817(A)	417	13.51	0.32	0.29	3.46	2.40
					811(A)	408	23.85	3.31	0.10	9.98	6.91
					812(A)	411	24.61	1.43	0.04	27.87	19.31
**IsiA3**	317.0	K108(F)	W28		102 (J)	404	18.46	2.01	0.28	3.53	2.44
		P104(F)	W29		101 (J)	404	17.13	0.17	0.04	26.44	18.32
		N-terminus (J)	E349		808(A)	417	22.80	1.29	0.05	19.52	13.53
**IsiA4**	371.3	K122(F)	K346	3.8	102(J)	417	12.51	0.03	0.04	23.41	16.22
		A125(F)	W29	3.8	102(J)	415	20.13	0.63	0.05	18.88	13.08
		L118(F)	Bcr421	4.1	101(J)	417	25.77	2.10	0.04	24.99	17.32
**IsiA5**	229.2	F310(B)	W28	3.7	841(B)	404	15.10	1.28	0.60	1.66	1.15
		T312(B)	W29	3.7	101(X)	417	23.53	1.45	0.05	20.97	14.53
		D485(B)	L309	4.3							
**IsiA6**	4.1				841(B)	417	18.46	1.29	0.18	5.50	3.81

*The lifetime (τ) and half-life ($t_{1/2}$) were defined as $\tau = 1/K_{DA}$ and $t_{1/2} = \ln 2/K_{DA}$, respectively.

References

1 Thompson, J. D., Higgins, D. G. & Gibson, T. J. CLUSTAL W: improving the sensitivity of progressive multiple sequence alignment through sequence weighting, position-specific gap penalties and weight matrix choice. *Nucleic Acids Res* **22**, 4673-4680, doi:10.1093/nar/22.22.4673 (1994).

2 Gouet, P., Robert, X. & Courcelle, E. ESPript/ENDscript: Extracting and rendering sequence and 3D information from atomic structures of proteins. *Nucleic Acids Res* **31**, 3320-3323, doi:10.1093/nar/gkg556 (2003).
