# Supplementary material for: Structure of a cyanobacterial photosystem I surrounded by octadecameric IsiA antenna proteins
Source: Commun Biol. 2020 May 11;3:232. doi: 10.1038/s42003-020-0949-6 (PMC7214436; doi:10.1038/s42003-020-0949-6)
Supplement: Supplementary file 3 — Description of Additional Supplementary Files [file 42003_2020_949_MOESM3_ESM.pdf]

Description of Additional Supplementary Files

File Name: **Supplementary Data 1**
Description: Source Data in Fig. 5 in Excel format
